# Molecular signatures in the progression of COVID-19 severity

**DOI:** 10.1038/s41598-022-26657-2

**Published:** 2022-12-21

**Authors:** Ronika De, Rajeev K. Azad

**Affiliations:** 1 Department of Biological Sciences and BioDiscovery Institute, University of North Texas, Denton, TX 76203 USA; 2 Department of Mathematics, University of North Texas, Denton, TX 76203 USA

**Keywords:** Computational biology and bioinformatics, Molecular biology, Diseases

## Abstract

SARS-CoV-2 is the causative agent of COVID-19 that has infected over 642 million and killed over 6.6 million people around the globe. Underlying a wide range of clinical manifestations of this disease, from moderate to extremely severe systemic conditions, could be genes or pathways differentially expressing in the hosts. It is therefore important to gain insights into pathways involved in COVID-19 pathogenesis and host defense and thus understand the host response to this pathogen at the physiological and molecular level. To uncover genes and pathways involved in the differential clinical manifestations of this disease, we developed a novel gene co-expression network based pipeline that uses gene expression obtained from different SARS-CoV-2 infected human tissues. We leveraged the network to identify novel genes or pathways that likely differentially express and could be physiologically significant in the COVID-19 pathogenesis and progression but were deemed statistically non-significant and therefore not further investigated in the original studies. Our network-based approach aided in the identification of co-expression modules enriched in differentially expressing genes (DEGs) during different stages of COVID-19 and enabled discovery of novel genes involved in the COVID-19 pathogenesis, by virtue of their transcript abundance and association with genes expressing differentially in modules enriched in DEGs. We further prioritized by considering only those enriched gene modules that have most of their genes differentially expressed, inferred by the original studies or this study, and document here 7 novel genes potentially involved in moderate, 2 in severe, 48 in extremely severe COVID-19, and 96 novel genes involved in the progression of COVID-19 from severe to extremely severe conditions. Our study shines a new light on genes and their networks (modules) that drive the progression of COVID-19 from moderate to extremely severe condition. These findings could aid development of new therapeutics to combat COVID-19.

## Introduction

A novel coronavirus, SARS-CoV-2, the most recent of Sarbecoviruses, was first identified in Wuhan, China in December 2019^1^ and is responsible for causing the global respiratory illness COVID-19 in over 642 million people and has killed over 6.6 million people^1,2^. This viral pandemic has affected millions of people around the globe. Viruses being obligate parasites evade the host cell immunity by producing antigenic peptides as well as other proteins using the host cell’s machinery. To enable the functioning of these processes, an intricate series of interactions between the host and virus is required^3^. To gain insights into the genes and pathways involved in the COVID-19 pathogenesis and host defense, the host response against this pathogen is being studied by scientists worldwide. Although some of the genes and pathways identified in hosts during pathogenesis are common across pathogens, each viral infection generates a unique transcriptional profile^4^ with the expression of several genes or biological pathways during an infection phase.

At present, the host response to the SARS-CoV-2 infection is not well-understood. Advances in high-throughput sequencing technologies have enabled gaining insights into the perturbations in the expression of different genes and biological pathways that result in a wide range of clinical manifestations in hosts. Although several studies have focused on differential gene expression during the SARS-CoV-2 infection^5–11^, these analyses are based mainly on the criteria of expression fold-change and statistical significance for identifying. It is recognized that many of the lowly expressed genes may also be biologically or medically significant but may get filtered out due to their not meeting the preset criteria. Identifying false negatives is a non-trivial problem; it could be tempting to relax the criteria to identify even more true positives but there is an inherent risk of incurring false positives by this approach. In disease conditions such as COVID-19, it is important to reliably infer genes that are playing certain roles in the conditions. We posit that this is possible by invoking multiple complementary approaches, including the classical “gene-by-gene” approach and systems-level approach that could emphasize on gene interactions. Clearly, there is a pressing need of a systems-level approach to investigate networks of host genes regulated during different stages of COVID-19. To address this, a systems-level differential gene expression analysis was performed to decipher genetic factors implicated in various severity conditions of the SARS-CoV-2 infection. The overarching goal of this study is to augment our understanding of genes, as well as pathways, that play diverse roles during various clinical manifestations of COVID-19 pathogenesis, ranging from moderate to extremely severe infections including conditions that require patients to be admitted to intensive care units (ICUs) or put on life support.

**Figure 1:**
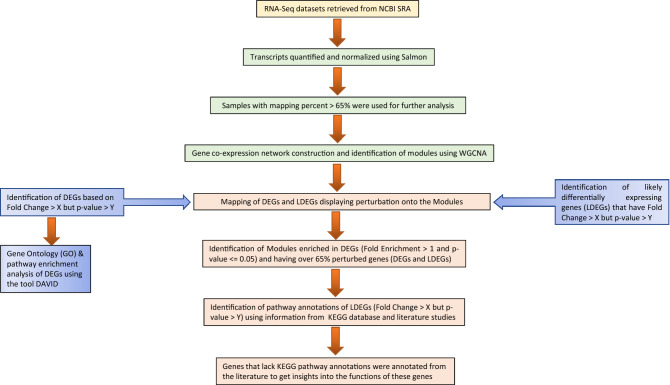
Schematic diagram of the gene co-expression network-based pipeline. Step(s) involved in data download are represented in yellow. Data processing steps are represented in green. Differential gene expression, likely differential gene expression and functional annotation enrichment analysis steps are represented in blue. Data analysis steps are represented in orange. X: cut-off for fold-change and Y: cut-off for p-value.

Most of the previous transcriptomic studies based on bulk RNA-Seq focused on various stages of the infection but not on the progression of the infection from moderate to extremely severe condition^10–16^. Thus, the information on genetic factors involved during the progression of COVID-19 from moderate to severe and then to extremely severe infection is lacking. To gain new insights into the progression of COVID-19 severity at the mechanistic and regulatory levels, we developed a novel gene co-expression network based pipeline (Fig. 1) that uses transcriptomic (RNA-Seq) data obtained from experiments on various tissues including lung, blood, and pancreatic organoids created from human embryonic stem cells (hESCs) to first construct a COVID-19 gene co-expression network and then identify modules enriched in differentially expressing genes (DEGs) in different COVID-19 severity stages. Specifically, given an expression dataset representing a COVID-19 severity stage, DEGs identified in this dataset based on both the fold-change and the statistical test are mapped onto the gene co-expression network and genes modules (clusters of genes tightly linked by co-expression in the network) that are significantly enriched in DEGs (p-value ≤ 0.05) are identified. Through this approach we identified not the DEG enriched modules that represent pathways or networks of functionally linked pathways that are differentially regulated during COVD-19 progression, but also, by association, several genes of interest in these modules that were elevated or depressed in the expression level by several fold yet were deemed non-significant by the statistical test used. Our analysis revealed several modules with abundance of such genes (including DEGs) that were interrogated to determine functions that were enriched in these modules using KEGG pathway database^17^ and literature studies. In what follows we describe our approach, results from our analysis, and discuss the implications of this study and future directions.

## Materials and methods

### Transcriptomic data analysis

We obtained the expression profile datasets for SARS-CoV-2 infected tissues, including lung cell lines A549, bulk peripheral blood mononuclear cells (PBMC), and pancreatic organoids created from human embryonic stem cells (hESCs) from National Center for Biotechnology Information (NCBI) GEO database (https://www.ncbi.nlm.nih.gov/geo/). The accession numbers of RNA-Seq datasets used for gene co-expression network analysis are GSE154613 and GSE152418. The transcripts from RNA-Seq data were derived using Salmon^18^ and transcript abundance estimates were obtained using the tximport^19^ package of R. Samples with reads covering greater than 65% of the human transcriptome in the alignment were used from further analysis (Table S1). We performed differential gene expression analysis (on GSE152418) using DESeq2^20^, which is different from the Z-score based expression analysis performed in the original study^12^. Genes were catalogued as differentially expressed based on log_2_ fold-change ≥ 1 or log_2_ fold-change ≤ − 1, and false discovery rate (FDR) adjusted p-value ≤ 0.05.

### Functional annotation enrichment analysis

As functional annotation enrichment analysis was not performed in the original study^12^, we performed gene ontology (GO) and pathway enrichment analyses on DEG sets to understand their functional significance using DAVID (https://david.ncifcrf.gov/). GO terms providing information about cellular localization, molecular function, and biological process were obtained. GO terms and biological pathways that were significantly overrepresented (p-value ≤ 0.05) in a DEG set were identified as enriched.

### Gene co-expression network construction

We constructed a matrix (21,423 rows and 200 columns) of normalized transcript expression values, with rows representing transcripts and columns representing experiments. This expression matrix was then imported to the Weighted Gene Correlation Network Analysis (WGCNA)^21^ package and a gene co-expression network was constructed using blockwiseModules function to generate a signed network under a soft-thresholding power of 10, minimum module size of 20, merge cut height of 0.15, and maximum block size of 40,000. Following network construction, gene modules were generated based on a hierarchical clustering approach using WGCNA. The gene modules are comprised of genes that are highly correlated in their expression under many different conditions, as represented in the different RNA-Seq datasets used. The co-expression network is comprised of 21,170 nodes and 7,574,232 edges. Each node represents a gene, and each edge represents a connection or association between the nodes (genes). The association between nodes is quantified based on the topological overlap value, ranging from 0 to 1, taking into consideration both the expression profile similarity, and the similarity of relationships each node has with all other nodes. A WGCNA network is fully interconnected, but each connection is weighted differently. Only genes that fall into module 0 are disconnected from other genes in the network. The large size of the network didn’t allow visualization using Cytoscape^22^ and therefore, we removed weak edges based on their weights. Edges with weights < 0.19 were removed to enable visualization using Cytoscape. The resultant network that could be visualized with Cytoscape consists of 15,696 nodes and 4,319,901 edges. Note that the aforementioned steps were performed to trim the network to enable visualization. All downstream analyses were performed with the complete (untrimmed) network.

### Mapping of DEGs onto the network

DEGs from an RNA-Seq dataset representing a SARS-CoV-2 infected tissue or organ were mapped onto the gene network. This provided information about DEG containment of each module in the network. The DEG containment of some modules range from none to a few DEGs, while other modules consist of a large number of DEGs.

### Identification of modules enriched in DEGs

Modules enriched in differentially expressing genes were determined by assessing the statistical significance of fold enrichment of DEGs in modules with fold enrichment > 1. This was accomplished by performing the Fisher test and the modules with p-value ≤ 0.05 were deemed significantly enriched. We did not use higher threshold for fold enrichment to avoid losing any modules that could be potentially important under certain severity condition of COVID-19.

### Identification of likely differentially expressing genes (LDEGs)

We considered DEG enriched modules and because of the enrichment, we deemed these modules that could likely be representing pathways or subnetworks of pathways to be differentially expressing. However, these modules also harbor genes that were deemed non-DEG by the statistical test. Because of their residing within the DEG enriched modules, it is plausible that many of these genes may be participating in the same biological processes as the DEGs, and thus could be on same pathways as DEGs. Based on this premise, we posit that among these genes, those with fold-change of two or more are likely differentially expressing genes (LDEGs) but were missed as they lie below the detection threshold used by the standard statistical test. We further selected those DEG enriched modules that have a large majority (over 65%) of the genes DEGs or LDEGs. We investigated LDEGs in these modules for their potential roles in COVID-19 pathogenesis, specifically in the progression of COVID-19 severity.

### Pathway annotation for LDEGs

LDEGs were annotated for pathways they are part of using the KEGG pathway database as well as based on literature studies. This analysis sheds light on the functions of LDEGs and help us decipher if LDEGs are parts of some known pathways or play roles in yet unknown pathways.

To summarize, we first performed differential gene expression analysis to catalog genes that express differentially during different severity conditions of COVID-19. Although, this provided a list of genes of interest, information regarding pathways or networks of pathways in which these genes function was elusive. Mapping differentially expressing genes onto known pathways will identify differentially expressing pathways but will miss yet unknown pathways that differentially express during different COVID-19 states or conditions. We therefore constructed a gene co-expression network using transcriptomic data from SARS-CoV-2 infected tissues, namely, lung, blood, and pancreatic organoids, and identified modules that contain genes that are densely linked to each other because of their co-expression. Genes within the modules that represent pathways or networks of pathways co-express under many different conditions or across different tissues in COVID-19. Mapping of differentially expressing genes from a COVID-19 expression dataset onto the modules allowed to decipher pathways, both known and unknown, that associate with a certain state of COVID-19 represented by the dataset. Specifically, following mapping of DEGs, modules enriched in DEGs were identified and further analyses were performed to identify LDEGs that are prone to be missed by standard protocols of expression analysis. This enabled identification of differentially expressing pathways or networks of pathways, represented by modules in the network, during various stages of COVID-19. Functional annotation (GO and KEGG pathway) enrichment analysis of modules enriched in DEGs was performed for identification of biological processes and pathways involved in various clinical manifestations of COVID-19.

## Results and discussion

### Differential gene expression analysis of human cells infected with SARS-CoV-2

**Figure 2:**
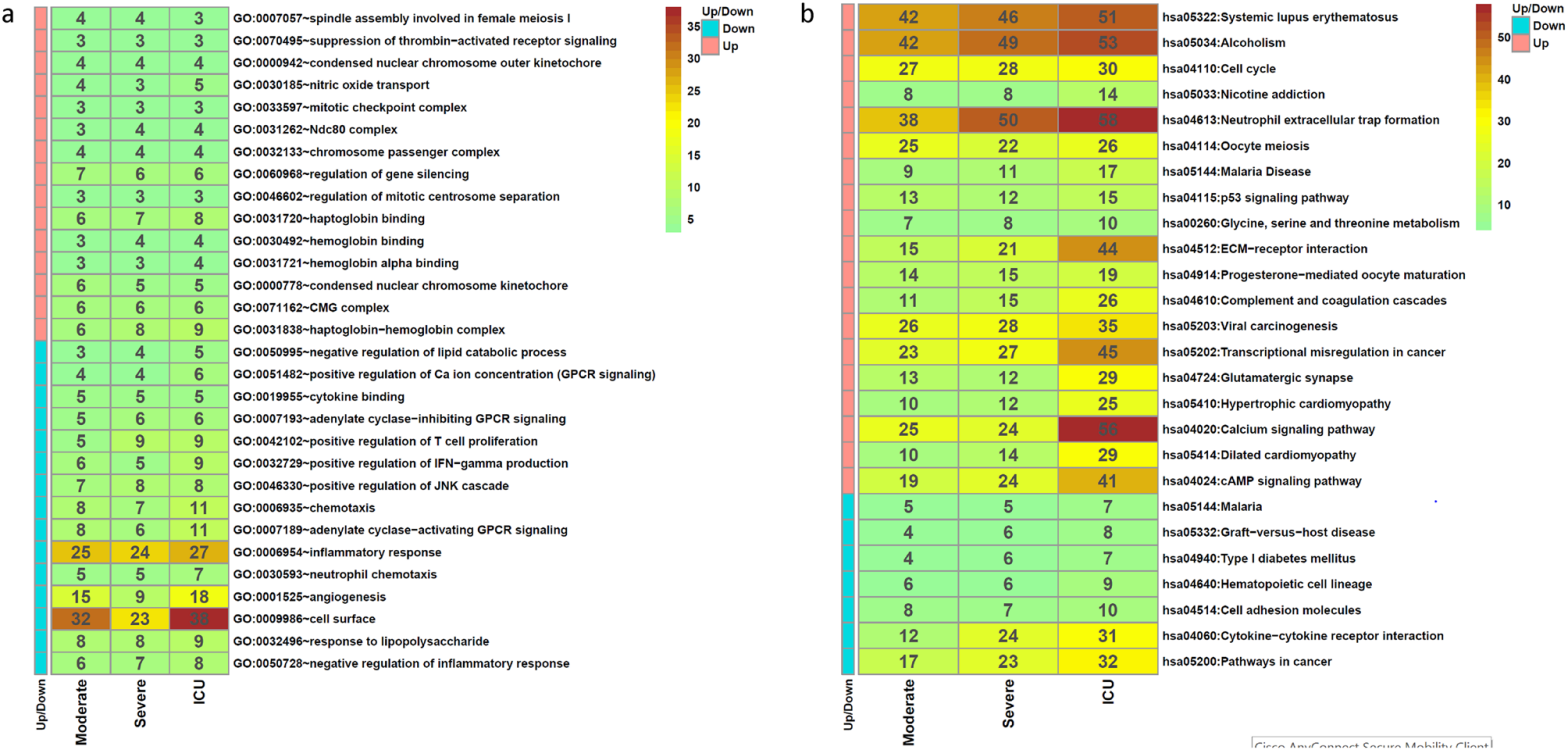
Heatmap of (**a**) gene ontology (GO) terms and (**b**) biological pathways enriched during moderate, severe and extremely severe (ICU) COVID-19. The values in each box of the heatmap represents fold enrichment of the GO terms and pathways for moderate vs. healthy, severe vs. healthy and extremely severe (ICU) COVID-19 infection vs. healthy. The upregulated and downregulated pathways are indicated by the color bar at the left of heatmap. The heatmaps were generated using the ‘pheatmap’ (version 1.0.8)^81^ package of R (version 4.0.0)^82^ available at http://cran.nexr.com/web/packages/pheatmap/index.html.

To understand the host response to the SARS-CoV-2 infection, we analyzed transcriptomic data from PBMCs of patients infected with SARS-CoV-2 and healthy controls retrieved from NCBI SRA (SRP267176) or NCBI GEO (GSE152418). The SARS-CoV-2 infected patients showed a wide range of clinical manifestations including moderate, severe and extremely severe (ICU) symptoms of COVID-19. As the authors in the original study^12^ assessed the relative contributions of multiple factors (age, gender, severity, and days post onset of disease symptoms) to the variance in gene expression between COVID-19 patients and healthy individuals, here we focused on assessing different biological pathways regulated by the DEGs in patients with moderate COVID-19 symptoms (Moderate vs. Healthy), severe COVID-19 symptoms (Severe vs. Healthy), and extremely severe (ICU) symptoms (ICU vs. Healthy). Using our approach for differential gene expression analysis (see Methods), we identified even more DEGs than reported by the previous study in all the three stages of COVID-19^12^. We found 1,540 DEGs in Moderate vs. Healthy, 1,496 DEGs in Severe vs. Healthy, and 3030 DEGs in ICU vs. Healthy (Table S2). To understand the biological processes and pathways affected during different stages of COVID-19 due to perturbed expression of genes, we performed functional annotation enrichment analysis for DEGs obtained from bulk RNA-Seq data, an important aspect that, however, was not probed in the original study^12^. We identified GO functions and biological pathways enriched during all stages of COVID-19 (Fig. 2). GO enrichment analysis revealed upregulation of genes encoding proteins that are known to be involved in nitric oxide transport such as hemoglobin alpha chain, beta chain and aquaporin-1. Although some studies have speculated that SARS-CoV-2 proteins destabilize hemoglobin beta chain^23,24^, a recent study has revealed that hemoglobin is not damaged by the viral proteins^25^. We posit that higher expression of hemoglobin and aquaporin-1 genes could possibly indicate their role in nitric oxide transport. As nitric oxide is involved in the regulation of vascular integrity by inhibiting platelet aggregation and coagulation^26^ and is, therefore, used as a therapy for COVID-19^27,28^, our analysis suggests that the upregulation of nitric oxide transport could be a host response against the infection and provides a scope for further investigation into the role of increased expression of hemoglobin and aquaporin-1 genes during COVID-19. We detected upregulation of genes encoding ALAS2, GLDC, CBS, and PSAT1 that are involved in glycine, serine, threonine metabolism. Recent studies have revealed higher glycine, serine, threonine metabolism in mild vs healthy^29,30^, severe vs healthy^30^, and severe vs mild^29^, but none of these studies considered samples from ICU patients with extremely severe COVID-19 infection. Our analysis highlights the dysregulation of glycine, serine, threonine metabolism in ICU patients as well. Pathway enrichment analysis revealed an increase in the number of perturbed genes involved in glycine, serine, threonine metabolism with the severity of COVID-19 infection (7 in moderate, 8 in severe, and 10 in ICU) as shown in Fig. 2. We found downregulation of genes playing a role in hematopoietic cell lineage including *CSF1R, CR1, CSF3R, IL1B, IL3RA, CD1D* in moderate, *KIT, IL5RA, TNF, IL1A, IL1B, ITGA3* in severe and *TNF, IL1A, IL1B, ITGA3, HLA-DOA, IL9R, CR2, CD8B, FCER2* in ICU patients. Unlike studies that found perturbation of hematopoietic system to be a marker of severe and fatal cases of COVID-19^31,32^, we found dysregulation of 6 genes involved in hematopoietic cell lineage in both moderate and severe infection, while 9 genes were perturbed in ICU indicating that hematopoietic system is dysregulated across different stages of COVID-19 with higher number of genes downregulated during the extremely severe infection.

We further performed differential gene expression and pathway analysis of the DEGs identified in patients with severe versus moderate COVID-19 symptoms (Severe vs. Moderate), and in extremely severe (ICU) versus severe patients (ICU vs. Severe), which were not investigated previously using RNA-Seq of PBMCs representing different COVID-19 stages^8–10,12^. This analysis was performed to gain insights into genes or pathways differentially regulated during the progression of this disease from the moderate to extremely severe condition. We identified 685 DEGs in Severe vs. Moderate and 344 DEGs in ICU vs. Severe comparisons (Table S2). We identified 27 conserved DEGs that were upregulated in both Severe vs. Moderate and ICU vs. Severe (Table S3). Some of these DEGs including *PF4V1* and *PPBP* function in cytokine-cytokine receptor interaction; *PROS1* and *SERPINB2* in complement and coagulation cascades; and *NOTCH3*, *ITGB3* and *THBS1* in cancer pathways. These results indicate that activation of cytokine mediated immune modulation as well as complement and coagulation pathways are key players involved in the progression of COVID-19 from moderate to severe and then to extremely severe condition. We identified 433 genes that were upregulated and 150 genes that were downregulated in Severe vs. Moderate but were not differentially expressed in ICU vs. Severe. Some of these upregulated genes include *ALOX5, TBXAS1, GGT1, LTA4H,* and *PTGES* involved in arachidonic acid metabolism; *IL1R2, CSF3R, IL13RA1, ACVR1B, IFNGR2, CXCL16, TNFSF13, TNFRSF10C,* and *CSF1R* involved in cytokine-cytokine receptor interaction; *C1QB, C1QA, SERPINA1, C5AR1, F5,* and *CR1* in complement and coagulation cascade; *SEMA3C, PLXNA2, UNC5B, PLXNC1, FES, SLIT1,* and *PLXNB2* in axon guidance; *TLR8, TLR4, TLR2, CD14,* and *TLR5* in toll-like receptor signaling pathway; *MGST1, GGT1, IDH1, PGD, ANPEP, GSTA4,* and *OPLAH* in glutathione metabolism; and *CANX, CTSS, CTSB, HLA-E,* and *HLA-B* in antigen processing and presentation. The downregulated genes include *IL5RA, CCL2, CCL8, IFNG, INHBA, ACVR1C, LIF, CCL25, XCL1, XCL2, IL7R, CXCL10, CSF1, TNF,* and *CCL4* involved in cytokine-cytokine receptor interaction; and *PDGFB, FGF9, HSPA8, NR4A1, DUSP5, DUSP10, DUSP2, HSPA6, CSF1, DUSP8,* and *TNF* involved in MAPK signaling pathway. These results suggest that pathways such as arachidonic acid metabolism, toll-like receptor signaling pathway, axon guidance, glutathione metabolism and cytokine-cytokine receptor interaction are critical for the progression of COVID-19 from the moderate to severe stage. As these genes were dysregulated only during the progression of COVID-19 from moderate to severe, they are potential biomarkers of the moderate to severe disease progression. Similarly, we detected 250 genes that were upregulated and 37 genes that were downregulated in ICU vs. Severe but were not differentially expressed in Severe vs. Moderate. Some of these upregulated genes include *GNG11, CXCL3, PF4,* and *ADCY6* involved in chemokine signaling pathway; *MPL, TNFSF4, CXCL3,* and *PF4* in cytokine-cytokine receptor interaction; *EHD3, EHD2, CAV2, PDGFRA, ASAP2, RUFY1, DNM3,* and *HLA-C* in endocytosis; and *TBXA2R, GUCY1B1, MYLK, GP6, PTGS1, VWF, F2RL3, PRKG2, GUCY1A1, GP9, ADCY6, GP1BA, ARHGEF12,* and *GP1BB* in platelet activation. Some of the downregulated genes include *GZMB, SH2D1B, NCR3,* and *KLRC3* that have been implicated in natural killer cell mediated cytotoxicity. These results indicate increased endocytosis and reduced natural killer cell mediated cytotoxicity are critical to the progression from severe to extremely severe COVID-19. As these perturbed genes were uniquely associated with the progression of COVID-19 from severe to extremely severe condition, they are the potential biomarkers for severe to extremely severe COVID-19 progression.

*IL-6*, which encodes a cytokine, and was previously identified as an important marker for progression of COVID-19 by some of the studies^33–36^, was not found to be either differentially expressing or likely differentially expressing in our study, which could possibly indicate therapeutic interventions.

### Co-expression network analysis for identification of networks of COVID-19 associated genes

#### Network analysis for COVID-19 vs. healthy humans

**Figure 3:**
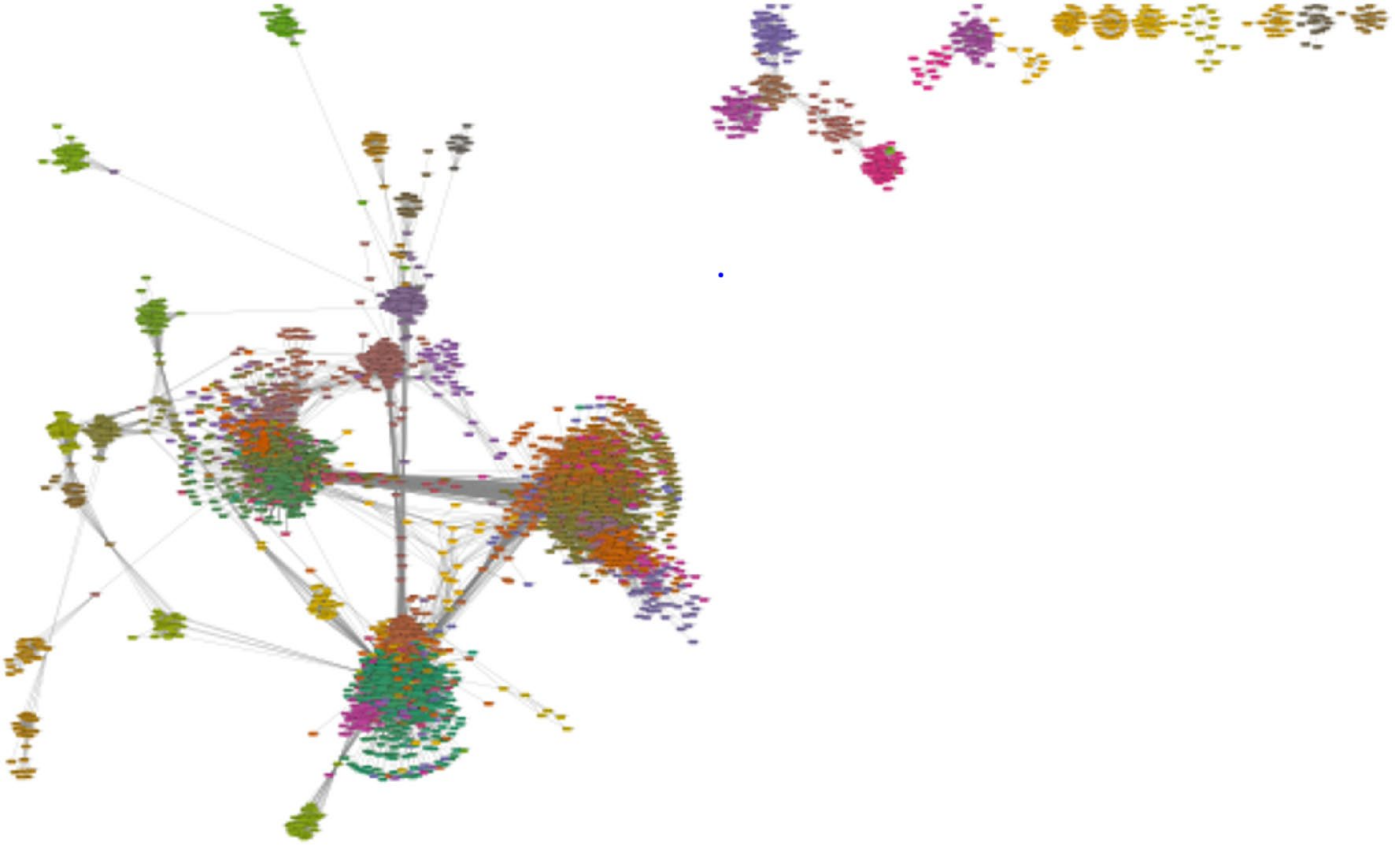
Gene co-expression network of SARS-CoV-2 infected humans. Gene modules are highlighted in different colors. Genes (nodes) are represented as small rectangular boxes and edges are represented by silver lines.

**Figure 4:**
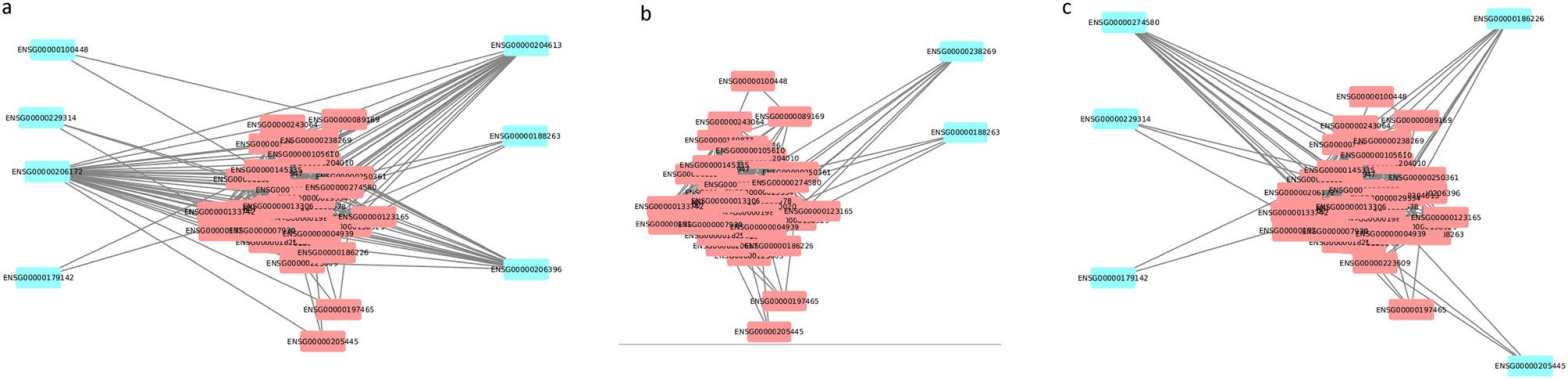
Likely differentially expressing genes (LDEGs) identified in Module 53 in (**a**) moderate versus healthy, (**b**) severe versus healthy, and (**c**) extremely severe (ICU) versus healthy. LDEGs are represented in blue color.

**Figure 5:**
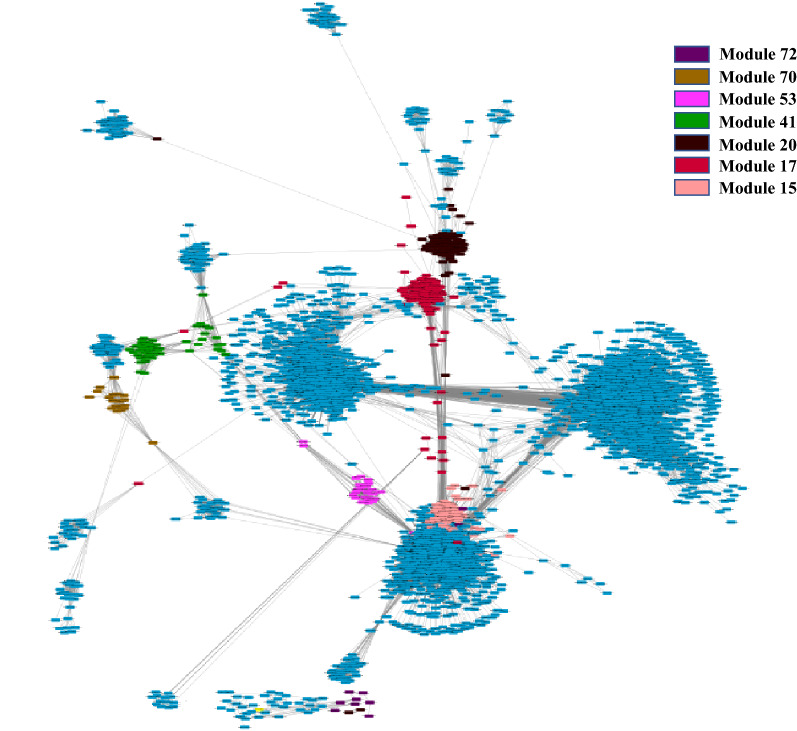
Modules of interest in gene co-expression network of SARS-CoV-2 infected humans are highlighted in different colors as indicated in the legend inset.

To uncover gene networks and pathways that play important roles during the SARS-CoV-2 infection, we took a systems approach, namely, performed weighted gene co-expression network analysis (WGCNA) of gene expression data obtained from the COVID-19 patients. First, a gene co-expression network for the human host was constructed (Fig. 3) using RNA-Seq datasets for SARS-CoV-2 infected humans by employing the network construction tool WGCNA (see Methods). A total of 77 gene modules were identified in this network (Table S4). The DEGs identified in moderate, severe, and extremely severe SARS-CoV-2 infections (all relative to healthy) were then mapped, in turn, onto the network to identify DEG enriched modules for each of these severity conditions. We examined further the DEG enriched modules that have a large majority of their genes (over 65%) either DEGs or LDEGs (Table 1, Table S5). As significantly high percentage of genes in these modules were deemed perturbed during SARS-CoV-2 infection, we posit that these modules play important roles in COVID-19. We found module 53 in moderate (Fig. 4a), severe (Fig. 4b), and extremely severe SARS-CoV-2 infected humans (Fig. 4c), with a large majority (over 65%) of genes either DEGs or LDEGs (relative to healthy) in this enriched module. We also identified another module enriched in DEGs (module 15) in extremely severe COVID-19 infection (relative to healthy) with over 65% of genes either DEGs or LDEGs (Fig. 5). To understand the roles of these modules (15 and 53) in different stages of COVID-19, we performed pathway enrichment analysis of the modules (Table S6). We identified enrichment of platelet activation, olfactory transduction, hematopoietic cell lineage, arachidonic acid metabolism, and ECM-receptor interaction in module 15. As platelet activation, impairment of olfactory transduction, dysregulation of hematopoietic cell lineage and arachidonic acid metabolism were previously associated with COVID-19^31,32,37–41^, we posit that module 15 is critical in enhancement of disease severity leading to extremely severe COVID-19. In module 53, we identified enrichment of amino acid (glycine, serine, threonine) metabolism. A recent study has revealed deregulation of amino acids involved in one-carbon metabolism such as glycine, serine and threonine during COVID-19^42^. As module 53 is conserved across all stages of COVID-19 and is involved in a pathway associated with COVID-19, it is highly likely that this module is critically involved in COVID-19 and needs to be investigated further to understand its role in augmenting the severity of this disease.

**Table 1:** Modules with over 65% genes either DEGs or LDEGs in moderate, severe, and extremely severe (ICU) SARS-CoV-2 infected humans relative to healthy humans.

Module	DEG count	LDEG count	Genes (DEGs + LDEGs) (%)	Disease severity	Enriched/Non-enriched
53	24	7	0.72	Moderate	Enriched
53	29	2	0.72	Severe	Enriched
15	144	43	0.89	ICU	Enriched
53	34	5	0.90	ICU	Enriched
70	1	17	0.72	Moderate	Non-enriched
17	10	129	0.70	ICU	Non-enriched
20	11	102	0.66	ICU	Non-enriched
41	8	38	0.65	ICU	Non-enriched
72	6	11	0.70	ICU	Non-enriched

Interestingly, we found one module in moderate COVID-19 (module 70, Fig. 5) and four modules in extremely severe COVID-19 (modules 17, 20, 41, 72, Fig. 5) that are not significantly enriched in DEGs but are comprised of over 65% genes that are either DEGs or LDEGs (Table 1, Table S5). Non-enrichment indicates these modules are replete with LDEGs. These five potentially important modules need to be investigated further to establish their roles in COVID-19. To get insights into the potential roles of these modules in COVID-19, we performed pathway enrichment analysis of the modules (Table S6). We identified enrichment of olfactory transduction, antigen processing and presentation, and natural killer cell mediated cytotoxicity in module 70. Previous studies have revealed dysregulation of olfactory transduction^37^, and antigen processing and presentation^43^ during COVID-19, therefore, it is highly likely that module 70 in involved in moderate COVID-19. In modules 17 and 41, we identified enrichment of olfactory transduction. In module 20, we found enrichment of olfactory and taste transduction pathways. As impairment of olfactory and taste transduction pathways were previously reported in COVID-19 patients^37,44,45^, we posit that pathways represented by modules 17, 41 and 20 are dysregulated during extremely severe COVID-19.

### Network enables identification of new molecular players otherwise deemed insignificant by standard statistical analysis

We examined LDEGs for their involvement in any known pathways using KEGG pathway database and the literature (Table S7). In our assessment of gene expression in moderate SARS-CoV-2 infected humans relative to the healthy humans, module 53 was found to harbor LDEGs that were elevated in expression, such as *MPIG6B* that functions in platelet activation and regulation^46^, TRIM10 involved in negative regulation of IFN type-1 signal transduction^47^, *CYP11B2* involved in aldosterone biosynthesis, as well as a potentially downregulated LDEG, *ORM1*, that is known to be involved in immune modulation and lysosome. Previous studies have revealed the role of aldosterone in stimulation of macrophage infiltration and inflammation^48,49^. As immunological dysregulation^31,50,51^, sustained inflammation^52^, and platelet activation^38^ were revealed to be associated with COVID-19 therefore, these LDEGs could indeed be significant players in moderate COVID-19, as uncovered by our network-based analysis. Functional annotation of *IL17REL* was not available in KEGG database and was also not found in the literature. This gene is not associated with any known pathway but could be playing some yet obscured role during the moderate clinical manifestation of COVID-19. Similarly, comparison of severe COVID-19 with normal revealed LDEGs in module 53 that were elevated in expression such as, *PAGE2B* and *IL17REL*, however, they lack functional clarity as KEGG database and literature studies don’t link them to known pathways in the human. Further studies may reveal their roles in severe COVID-19.

Comparison of extremely severe COVID-19 with normal revealed 41 LDEGs with increased expression levels in module 15, which are known to be involved in one or more of these processes or functions based on KEGG database or the literature studies: cancer pathways, olfactory signal transduction, platelet activation, antigen processing and presentation, osteoclast differentiation pathways (see Table S7 for the list of these LDEGs). As these pathways have been reported to be activated in COVID-19^38,53–59^, these statistically non-significant genes could indeed be clinically significant and likely involved in extremely severe COVID-19. Several other LDEGs in this module, namely, *TSPYL6, DAZ1, KPRP, SPATA31D5P, GSTT4, REXO1L2P, SCGB1C2, LOC105371458* and *PRAMEF10* lacked functional clarity. In module 53, potentially upregulated LDEGs include *CTSG* involved in immune modulation and lysosome, *FCAR* involved in phagosome, and *CYP11B2* involved in aldosterone biosynthesis. Impairment of lysosomal pathway is one of the characteristics of SARS-CoV infection^60–62^, which is genetically similar to the novel coronavirus SARS-CoV-2. A recent study has revealed aberrant upregulation of lysosomal pathway during COVID-19^63^, thus genes involved in autophagy such as *CTSG* and *FCAR* could indeed be expressing differentially during extremely severe COVID-19. Several studies have revealed the role of immune dysregulation and chronic inflammation in COVID-19^31,50,52^, which gives further credence to our network driven inferences. These newly discovered genes likely associate with the extremely severe manifestation of COVID-19 and need to be further investigated.

### Co-expression network analysis for the identification of networks of genes involved in the progression of COVID-19 from moderate to severe to extremely severe infection

**Figure 6:**
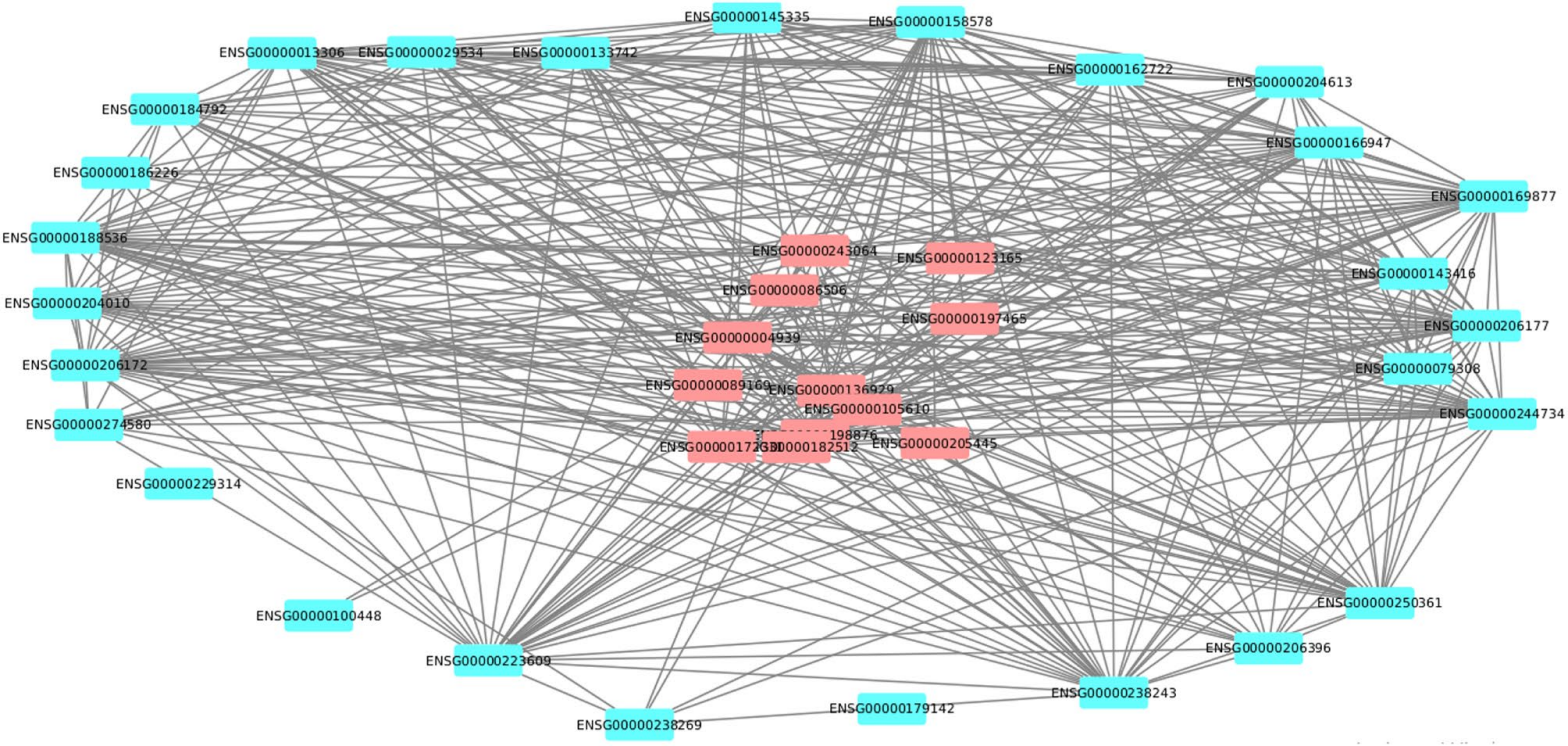
Likely differentially expressing genes (LDEGs) identified in Module 53 in extremely severe (ICU) vs severe COVID-19. LDEGs are represented in blue color.

To understand the roles of different genes, and pathways or networks of pathways, in the progression of COVID-19 from moderate to severe and then to extremely severe, we determined the modules enriched in DEGs and additionally, those comprised of over 65% DEGs or LDEGs (Table S5) for severe SARS-CoV-2 infection relative to the moderate infection and similarly for extremely severe SARS-CoV-2 infection relative to the severe infection. We first searched for DEG enriched modules that have over 65% DEGs or LDEGs in severe COVID-19 relative to the moderate infection. However, no such module signifying the progression of the disease from moderate to severe was identified. Interestingly, we identified a gene module (module 70) that is not enriched in DEGs from the severe COVID-19 stage (relative to the moderate) but had a large majority (over 65%) genes that are either DEGs or LDEGs (Fig. 5, Table 2). To identify pathways represented by this module that could be potentially associated with the progression of COVID-19 from moderate to severe, we performed pathway enrichment analysis of this module (Table S6). We found enrichment of pathways that were previously associated with COVID-19 such as olfactory transduction^37^, antigen processing and presentation^43^, and natural killer cell mediated cytotoxicity in this module. We also identified genes that were not previously implicated in COVID-19 such as *POTEG, LAIR2,* and *ZCCHC13* involved in cell cycle and cancer regulatory pathways^64–66^. We further identified 4 genes in this module that are not associated with any known pathways (Table S7). Our result indicates that this module is likely associated with the progression of COVID-19 from moderate to severe as it is enriched in COVID-19 associated pathways. It could be thus of interest to probe the roles of genes in this module that have not yet been implicated in COVID-19 (Table 3) as our module-based analysis associate these genes with COVID-19 implicated genes. We identified two DEG enriched modules (15 and 53) in extremely severe COVID-19 (relative to severe COVID-19), with a large majority (over 65%) of genes either DEGs or LDEGs (Fig. 5). Module 15 was also found to be enriched in DEGs in ICU patients (compared to healthy individuals), which further emphasizes on the importance of this module in COVID-19 pathology. LDEGs identified in module 53 (Figs. 4, 6) could be potentially important in COVID-19 pathogenesis as this module was found to be conserved across the three stages (moderate, severe, and extremely severe) of the infection, i.e., was found to be enriched in DEGs in each of these stages of COVID-19 infection (relative to healthy). Using information from the KEGG pathway database, as well as from literature studies, we found that many LDEGs identified for extremely severe vs. severe are indeed components of pathways implicated in COVID-19 including platelet activation, olfactory transduction, antigen processing and presentation, arachidonic acid metabolism, amino acid metabolism, and hematopoietic pathways (Table S7)^31,38–41,54,67–69^. This suggests that these genes could likely be involved, as parts of the aforementioned pathways, during the progression of COVID-19 from severe to extremely severe condition. We identified 25 LDEGs and 2 LDEGs that are not associated with any known pathway in modules 15 and 53 respectively (Table S7). Further follow-up studies are needed to decipher the roles of these LDEGs in the severe to extremely severe COVID-19 progression. These results highlight the power of network-based approach in discovering novel genes or pathways involved in COVID-19 pathology, addressing issues with traditional statistical approaches that may miss a substantial number of biologically significant factors. A comprehensive understanding can be gained by using both approaches in combination, as highlighted by our study on COVID-19.

**Table 2:** Module with over 65% genes displaying perturbed expression in severe SARS-CoV-2 infected humans relative to moderate and extremely severe SARS-CoV-2 infected humans relative to severe.

Module	DEG count	LDEG count	Genes (DEGs + LDEGs) (%)	Disease progression	Enriched/Non-enriched
15	77	69	0.69	Severe to extremely severe	Enriched
53	3	27	0.69	Severe to extremely severe	Enriched
70	2	18	0.8	Moderate to severe	Non-enriched

**Table 3:** LDEGs identified in module 70 for severe versus moderate COVID-19 comparison. These LDEGs, depleted in expression level in severe COVID-19 patients (relative to moderate COVID-19 patients), have not yet been implicated in COVID-19 pathogenesis.

Gene ID	Pathways
ESG00000171209	Acts as molecular chaperone and stabilizes caesin micelles
ENSG00000187537	Involved in regulation of cell cycle and apoptosis in tumor cells
ENSG00000187969	Modulates of ATK/ERK/c-MYC/CDK pathway during cell cycle progression in cancer cells
ENSG00000214237	Not associated with any known pathways
ENSG00000229292	Not associated with any known pathways
ENSG00000229493	Antigen presentation and T-cell stimulation
ENSG00000232629	Antigen presentation and T-cell stimulation
ENSG00000275621	Not associated with any known pathways
ENSG00000277335	Involved in cancer regulation
ENSG00000283463	Not associated with any known pathways

Note that the metadata of the SARS-CoV-2 infected patients used in this study (GSE152418) does not include information about any pre-existing conditions or comorbidities with COVID-19. Several studies have reported that COVID-19 patients with certain pre-existing diseases or conditions, such as cardiovascular conditions (including hypertension), cancer, diabetes, immunosuppression, lung disease, and neurological diseases, are at a greater risk of developing severe infection as well as at a heightened risk of dying due to the SARS-CoV-2 infection^70–79^.

The transcriptomic data used for differential gene expression analysis was taken from a previous study which was published in August 2020^12^ when the dominating strain was the initial SARS-CoV-2 identified in China, before the emergence of the alpha variant of SARS-CoV-2, which was first reported in September 2020 in Great Britain by the World Health Organization (WHO)^80^. Following the emergence of the alpha variant, various other variants of SARS-CoV-2, including beta, gamma, delta and omicron, have been reported by WHO^80^. With the emergence of these new variants and availability of relevant expression data, future studies could focus on identifying genes and pathways involved in pathogenesis and progression of COVID caused by these variants using network based methodologies, similar to the systems-level approach used in this study. Comparison of differentially regulated genes and pathways in humans afflicted by COVID caused by different variants will shed further light on molecular processes that are differentially impacted by SARS-CoV-2 variants.

## Concluding remarks

Conventional protocols for differential gene expression analysis use expression fold-change and statistical significance test to identify genes that are expressing differentially. Our study highlights the importance of a novel gene co-expression network-based approach to decipher biologically significant entities such as differentially regulated genes or modules that could fall below the detection threshold established in the traditional protocols for analyzing differential gene expression. Our network-based pipeline could be used in tandem with a conventional differential gene expression analysis pipeline to comprehensively identify genes or pathways differentially expressing under certain conditions, e.g. COVID-19 pathogenesis and progression of the disease.

Gene co-expression network analysis was performed to identify pathways and networks of pathways represented by modules, which play important roles in different stages of COVID-19, as well as in the progression of the disease from moderate to extremely severe infection. This systems-level analysis also helped to discover novel genes involved in the host defense or COVID-19 pathogenesis, which couldn’t be detected using standard protocols for differential gene expression analysis. Some of these genes encode hypothetical or yet uncharacterized proteins that have not yet been associated with known pathways but could be involved in COVID-19 pathogenesis and progression or potentially aiding the host defense. Our pipeline to identify modules enriched in genes differentially expressing during different stages of COVID-19 provides insights into key genes and pathways that might be playing important roles in COVID-19 pathogenesis and the host defense. Future studies could prioritize genes or pathways deemed significant in COVID-19, which may lead to new interventions to mitigate the symptoms or towards much needed novel therapeutics.

## Supplementary Information

Supplementary Tables.

## Data Availability

The NCBI GEO accession numbers of RNA-Seq datasets used for gene co-expression network analysis are GSE154613 and GSE152418. All other associated data and source codes have been made publicly available at the project’s GitHub site: https://github.com/Ronika19/COVID-19_Human_Network.
